# The Early Years Home Learning Environment – Associations With Parent-Child-Course Attendance and Children’s Vocabulary at Age 3

**DOI:** 10.3389/fpsyg.2020.01425

**Published:** 2020-06-30

**Authors:** Anja Linberg, Simone Lehrl, Sabine Weinert

**Affiliations:** ^1^Department of Social Monitoring and Methodology, Deutsches Jugendinstitut, Munich, Germany; ^2^Psychology I - Developmental Psychology, University of Bamberg, Bamberg, Germany

**Keywords:** home learning environment, socio-economic background, parent-child course, vocabulary development, longitudinal study, parent-child-interaction

## Abstract

Although many studies investigated the effects of the home learning environment (HLE) in the preschool years, the constructs that underlie the HLE in the years before the age of three and its effects on language development are still poorly understood. This study therefore investigated the dimensionality of the HLE at age two, its relation to the attendance of low threshold parent-child-courses, and its importance for children’s vocabulary development between age 2 and 3 years against the background of differing family background characteristics. Using data from 1,013 children and their families of the Newborn Cohort of the German National Educational Panel Study, structural equation modeling analyses showed that (1) quantitative and qualitative aspects of the early HLE, i.e., the frequency of stimulating activities, and the quality of parent-child-interactions should be differentiated; (2) that family background variables are differentially associated with the HLE dimensions and (3) that attendance at parent-child courses enriches both aspects of the HLE which in turn (4) are related to the children’s vocabulary development. Our results highlight the need to differentiate aspects of the early HLE to disentangle which children are at risk in terms of which stimulation at home and the possibility to enrich the HLE through low threshold parent-child courses.

## Introduction

Research consistently documents the great importance of early language skills for children’s later language and literacy development and overall school success (Duncan et al., 2007; Marchman and Fernald, 2008; Bornstein et al., 2016). Because of their major importance for reading comprehension (Storch and Whitehurst, 2002) language skills are often described in the framework of “emergent literacy skills,” which captures skills preceding formal reading in school, including code-related skills such as phonological awareness and letter knowledge, as well as language related skills, such as vocabulary and grammatical skills (Teale and Sulzby, 1986; Whitehurst and Lonigan, 1998; Sénéchal et al., 2001; Pinto et al., 2017). From early on children differ widely in their vocabulary knowledge with significant disparities associated with their socio-economic background (Hoff, 2003; Brooks-Gunn and Markman, 2005; Dubowy et al., 2008; Hindman et al., 2008; Fernald et al., 2013). These early differences in vocabulary can partly be traced back to experiences in the learning environment at home, which might be especially important during the early years, when vocabulary starts to grow quickly (Hart and Risley, 1995). Families stimulate children’s language development through various activities and interactions during infancy (NICHD Study of Early Child Care, 1999; Attig and Weinert, 2019). Several factors have been suggested as being of particular relevance: the frequency of stimulating activities, such as joint picture book reading, singing songs, or telling rhymes to a child (Rodriguez and Tamis-LeMonda, 2011), and the overall quality of interaction behavior in parent-child interactions, especially how sensitively the caregiver responds to the child’s signals (Ainsworth et al., 1974). The study by Rodriguez and Tamis-LeMonda (2011) for instance, revealed that children with more supportive learning environments in terms of quantity and quality of stimulation within the 1st year of life show comparatively larger vocabulary than children with less stimulation or later-starting stimulation.

Given the impact of the quality of parent-child interactions and the frequency of joint activities in the early home learning environment (HLE) on vocabulary development, enriching the HLE from early on seems to be important. Comprehensive interventions starting early in life have been demonstrated to be effective both with regard to the HLE and child development (Baker et al., 1998; Zigler et al., 2008; Nievar et al., 2011; Sama-Miller et al., 2017; Schaub et al., 2019). Although often not integrated within a broader framework of early intervention, parent-child courses are designed to give parents practical information regarding the nutrition or education of their toddlers and thus might enrich the HLE and stimulate child development.

Thus, although the importance of the very early years in children’s development is emphasized throughout the research literature, only marginal attempts have been made to investigate the dimensionality of the HLE before the age of three and the importance of such possible different aspects of early HLE and parent-child courses attendance for vocabulary development. In particular, it is unclear whether the HLE at that early age is composed of multiple, separable dimensions of quantitative, and qualitative aspects or whether it is unitary in nature and associated with similar family background characteristics.

The present paper therefore investigates the differential contribution of various HLE measures, including parent-child-course-attendance to vocabulary development against the background of varying family background characteristics.

## The Home Learning Environment of Toddlers

Following the bio-ecological model of human development, child development takes place in various contexts, and the characteristics of these contexts as well as how the child shapes them are important (Bronfenbrenner, 1999). In the first years of a child’s life the family is the most important learning environment, and processes in the family context, such as activities or interactions, are important engines of child development.

Of these processes, shared picture book reading is one of the most studied variables in research addressing the HLE in early childhood, and has been shown for decades to be of major importance in explaining differences in preschoolers’ language development (Bus et al., 1995; Mol et al., 2008; Flack et al., 2018). However, whether or not the important connection between sharing books with preschoolers and children’s language development can be transferred to the very early years has been less studied. The vast majority of parents report that they begin reading to their children when they are around 6 months of age (Debaryshe, 1993; Niklas et al., 2016). Research shows that joint picture book reading with infants and toddlers and the early onset of reading is linked to children’s vocabulary (Debaryshe, 1993; Karrass and Braungart-Rieker, 2005; Sénéchal et al., 2008; Niklas et al., 2016; Attig and Weinert, 2019). An intervention study by Karrass and Braungart-Rieker (2005) for instance, demonstrated that presenting 8 month old infants with picture books resulted in advanced vocabulary status at 12 and 16 months, although reading to 4 month olds did not have a similar effect. Furthermore, picture books have been used in a variety of word learning studies to teach new words to children. Research shows that children as young as 15 months learn new words from picture books after a single presentation (Ganea et al., 2009; Horst et al., 2011; Weisleder and Fernald, 2013; Montag et al., 2015). The mechanism behind the effect of sharing books with children on language development may be the provision of focused language input (Bruner, 1985). Besides reading to the child, there are other related activities that have also been shown to stimulate language acquisition such as singing songs or repeating nursery rhymes (Baker, 2013; Skwarchuk et al., 2014). In the following study we refer to the frequency of these language stimulating parent-child activities as a quantitative indicator or aspect of the HLE.

In the first years of children’s lives the HLE is not only characterized by these quantitative aspects, but also by the quality of children’s interactions with parents both during those activities as well as during daily routines such as feeding or diapering. We will refer to the quality of these interactions as the qualitative aspects or indicators of the HLE. The quality of interaction behavior can be differentiated into two main components: sensitivity and stimulation (Vallotton et al., 2017; Linberg, 2018). Sensitivity refers to the warm, accepting, prompt, and contingent responses parents provide in response to children’s affective, vocal, and gestural cues (Ainsworth et al., 1974; Shin et al., 2008). Stimulation refers to interactions and activities between parents and their child that promote the child’s cognitive and language development, e.g., by supporting them in exploring their environment, or by presenting them with stimulating materials and toys, or by using rich and varied language (Bradley et al., 2003). The quality of mother-child interactions during the first years of life has been shown to be positively associated with children’s vocabulary at the age of 30, 38, and 40 months (Nozadi et al., 2013; Wade et al., 2018). Even the interaction quality that children experience during their prelinguistic phase predicts language development later on. For example, Nicely et al. (1999) demonstrated that the quality of sensitive maternal responses to the signals of their 9-month-old children was associated with children’s language development at 21 months. Additionally, Tamis-LeMonda et al. (2001) have shown that infants experiencing high quality mother-child interactions during the first 2 years of their lives achieved milestones, such as first words or the vocabulary spurt, 4–6 months earlier compared to infants experiencing lower levels of quality.

However, variations in quality experiences at home have been widely shown to be associated with family’s socio-economic background (Bradley et al., 2003; Kluczniok et al., 2013). Especially maternal education, income poverty, and social resources (e.g., having a partner in the household) have repeatedly shown to be associated with access to books at home, the overall living conditions (e.g., crowding) and the frequency and quality of stimulating activities (Evans, 2006; Mistry et al., 2008, 2010; Lehrl, 2018). Due to the family investment and stress model (Conger and Donnellan, 2007) such associations might emerge because parents that do not suffer from low education, economic hardship or being a single parent, are better able to provide material resources, and experiences that promote children’s health and cognitive development, as they experience less parental distress, gather more information on child development, and as a consequence may show less disruptions in parenting behaviors and provide more stimulating HLEs. Previous studies have confirmed the significant association of the HLE and such indicators of the family’s socio-economic background, particularly for preschool-aged children (Foster et al., 2005; Forget-Dubois et al., 2009; Bojczyk et al., 2016; Lehrl et al., 2020). Studies with children at younger ages also document this relationship. For example, with regard to the quality of parent-child interaction (qualitative aspect) at toddler age studies demonstrate that more highly educated mothers react in a more sensitive and responsive way to their child’s signals such as vocalizations (Gudmundson, 2012; Mills-Koonce et al., 2016; Neuhauser, 2018) and use more complex and varied syntax and vocabulary (Hoff et al., 2002). Linberg et al. (2017) found that, in Germany, disparities in the quality of mother-child interactions are visible by maternal education as early as the age of 7 months. Studies considering both quality of parent-child interactions and the frequency of stimulating activities in a composite score also point to an association between maternal education, income as well as being a single parent and the overall HLE (Bradley et al., 2003; Lugo-Gil and Tamis-LeMonda, 2008; Rowe, 2008; Magnuson et al., 2009). Only a few studies have systematically differentiated qualitative and quantitative aspects. For example, Mistry et al. (2008) showed socioeconomic status (SES), as indicated by income, maternal education, and welfare receipt, to be related to quantitative and qualitative indicators of home stimulation whereas in the group of immigrant-parents the effects sizes were higher for the quantitative aspect (see also Mistry et al., 2010 for similar associations between a cumulative risk-index and HLE). However, in the group of monolingual children the effect sizes of SES on qualitative and quantitative HLE were nearly the same (Mistry et al., 2008). Thus, although there is some research evidence that family socio-economic background variables are associated with children’s HLE, there is a lack of research on the specific relations. We therefore focus more explicitly on the specificity of such indicators of socio-economic background and their relationship to children’s quantitative and qualitative HLE, and language development at specific points in early childhood.

## Parent-Child Course Attendance as Informal Support Systems

Building on the bio-ecological model, family system intervention models assume that families are embedded within various contexts, and that child development and parenting can be supported within those contexts (Dunst and Trivette, 2009; Anders et al., 2017). Besides highly structured formal support, which is often realized through home-visitation programs or structured programs (Baker et al., 1998; Zigler et al., 2008; Nievar et al., 2011; Sama-Miller et al., 2017; Schaub et al., 2019), opportunities for more informal social support networks could function as resources for meeting family concerns and needs (Campbell et al., 2002; Dunst and Trivette, 2009). Such informal opportunities can be out-of-home activities that parents select for their children through attending specific courses addressed to parents with toddlers, for example, baby swimming, the Prague Parent-Child Program (PEKiP), baby music, and other programmes that provide additional stimulating experiences. Although such generic – as distinguished from language and literacy specific – courses lack a coherent theoretical foundation and empirical evidence on whether they reach their aims, they might be an important source of support in fostering child development and/or parents’ parenting competencies through discussion of problems in child rearing with others. This interaction and exchange with other parents might serve as a social support system, which is a key determinant of parenting (Bollen, 1989) and its positive relation to parenting quality has been shown in various studies (Shin et al., 2006; Neuhauser, 2018). Group-based courses might also reduce emotional distress by coming into contact with parents with similar challenges. Results of a meta-analysis point to this, by showing that group-based training programmes for parents led to short and long-term improvements of emotional distress (Barlow et al., 2012) which in turn could increase positive parenting (McLoyd, 1990; Conger and Donnellan, 2007).

In Germany, nearly 50% of parents with young children (ages 0–3 years) attend courses of this kind (Mühler and Spieß, 2008). These courses, for which parents normally have to pay in order to attend, usually take place on a weekly basis for a limited time and are partly based on structured programs. They are designed to promote parent–child interaction, to directly foster children’s cognitive or motor development, to experience arts and music, or/ and to support the building of social networks among parents (Wilke et al., 2017). Mühler and Spieß (2008) showed that mothers report higher adaptation skills for their 3 year olds when attending such courses compared to mothers who did not attend courses, regardless of the educational level of the mother. Furthermore, Wilke et al. (2017) reported higher receptive vocabulary for 3 to 4 year old children who had attended such courses since birth. The concept of parent-child courses in Germany is, to some extent, comparable to the Sure Start Children’s Centers in England, especially to the “stay and play” services, although these are especially designed for families at risk and their services are of no cost (Sammons et al., 2015). Results from an evaluation study show that attending such “stay and play” services programmes is positively correlated with the toddler HLE (mean age of the children 14 months) and the preschoolers’ HLE (mean age of the children 38 months) (Hall et al., 2019). Furthermore, HLE changes from the toddler to the preschool phase were predicted by attending stay-and play services at 3 years of age, which in turn predicted less externalizing problem behavior. Thus, there is evidence that attending play-based courses might impact children’s development via HLE changes. This potential to enrich the HLE to foster children’s development has mostly been realized through structured programs (Baker et al., 1998; Zigler et al., 2008; Nievar et al., 2011; Sama-Miller et al., 2017; Schaub et al., 2019). However, how and whether attending parent-child courses affects children’s development via HLE changes has not been investigated so far, especially in the German context.

## The Present Study

Parents foster children’s language development through various activities and actions. Indicators of the HLE that have been demonstrated to be associated with children’s vocabulary may either focus mainly on quantitative aspects or on qualitative aspects of HLE. Quantitative indicators are targeted by the frequency of (language stimulating) joint activities of parents and children. Most prominently, shared book reading has been identified as a meaningful activity promoting children’s language development (Mol et al., 2008; Flack et al., 2018). In addition, joint activities such as singing songs or repeating nursery rhymes seem to be important. Qualitative indicators of the HLE focus on the quality of interactions the child experiences in the HLE. Here concepts such as the sensitivity of the parent to the child’s signals as well as the quality of stimulation behavior have been addressed (Ainsworth et al., 1974). As with the quantitative aspects of the HLE, these qualitative indicators have also been demonstrated to be associated with children’s language development (Nozadi et al., 2013; Wade et al., 2018). Yet, only few studies investigate both dimensions of the HLE, and their differential relation to child’s vocabulary: Whereas results of Rodriguez et al. (2009) indicate joint activities as well as maternal engagement in interactions being somewhat equally predictive for vocabulary at 24 months, Schmitt et al. (2011) reports only joint activity but not maternal sensitivity being related to vocabulary. In addition, study results show that both aspects differ according to socio-economic background variables. Depending on their socio-economic background, children have different experiences both with regard to qualitative as well as to quantitative aspects of their HLE, which might impact children’s language development (Hart and Risley, 1995).

However, most studies focused on only one or the other aspect of the HLE. Additionally, although the importance of quality has been demonstrated for children at preschool age, less is known about quality in the framework of HLE in the very early years in life.

Attempts to enrich the HLE in order to foster children’s language development have been made through various programmes (for an overview: Sama-Miller et al., 2017). However, these programmes are often cost-intensive and might have a high threshold for participation. More cost- and time-efficient are parent-child courses, which parents often attend with their children in the first years of a child’s life. As these courses also include information on stimulating activities and actions, they might contribute to the enrichment of the HLE in the very early years and thus could provide a way to reduce the effects of socio-economic background. However, studies focusing on parent-child courses in the context of enriching the HLE are still sparse.

Thus, the present paper investigates, (1) the dimensionality of the HLE at age two by distinguishing between a quantitative and a qualitative dimension of the HLE at this early age, and (2) by investigating the differential association of the different aspects with family socio-economic background variables, (3) whether parent-child courses substantially enrich the different aspects of the HLE, and (4) how these aspects of the HLE are associated with children’s vocabulary development between age two and three. We are assuming that quantitative and qualitative aspects of the HLE are two separate, however, associated dimensions. In line with Mistry et al. (2008) and the family stress model (Conger and Donnellan, 2007) we suspect indicators of socio-economic background, i.e., maternal education and risk of income poverty, as well as social resources, i.e., being a single parent, to be associated with quantitative and qualitative indicators of HLE. As some studies pointed in the direction of specifity effects (Klebanov et al., 1998; Kluczniok et al., 2013) we assume the relations to be specific in terms of family socio-economic background dimension as well as HLE dimension. However, according to the lack of research regarding the specifitiy of effects in terms of experiencing single aspects of socio-economic background and their association to differentiated HLE dimensions we cannot make clear assumptions.

**FIGURE 1:**
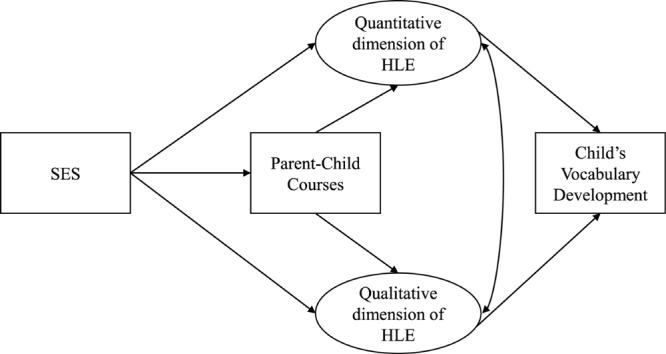
Proposed model on the relation between SES, the qualitative and quantitative dimension of home learning environment, parent-child-courses, and child’s vocabulary development.

Furthermore, we expect the attendance of parent-child-courses to enrich both dimensions of the HLE, as they could foster parenting skills through discussion of problems in child rearing as well as increase positive parenting by reducing emotional distress. However, associations might be more pronounced for the quantitative dimension, as most of these courses also include information on stimulating activities and actions. Additionally, based on previous study results we assume that both, the quantitative and a qualitative dimension of HLE, are associated with child’s vocabulary development (see Figure 1). The study carefully considers the direction of effects and potentially confounding variables.

## Methods

### Sample

To investigate our research questions, we used data of the Starting Cohort 1 – Newborns (SC1) of the German National Educational Panel Study (NEPS) (Weinert et al., 2016; Blossfeld and Roßbach, 2019). Based on a representative sampling frame, a nationwide sample of 3,500 infants born between February and June 2012 and their families were drawn, register-based (response-rate: 41%), and followed longitudinally (Weinert et al., 2016). We used the first four waves in our analyses and included all cases of mainly German-speaking families (i.e., who stated that their main interaction language at home was only or mostly German) with valid information on child’s vocabulary and HLE, which resulted in a total sample size of 1013 children. In wave 1 children were 7 months (*M* = 6.97, *SD* = 0.80), in wave 2, 13 months (*M* = 13.36, *SD* = 1.30), in wave 3, 26 months old (*M* = 26.49, *SD* = 1.19), and in wave 4, 38 months (*M* = 38.40; *SD* = 0.95; panel stability 71%). Table 1 presents an overview of measures and time-points of measurement used in the present study.

**TABLE 1:** Variables used in main analyses with waves.

	Wave 1	Wave 2^a^	Wave 3	Wave 4
Average age of child in months	7	13	26	38
Quantitative dimension of HLE	x		x	
Qualitative dimension of HLE	x		x	
Parent-child courses	x	x	x	
Child vocabulary		x	x	x
Socio-economic background	x			

*^*a*^By design in wave 2 the qualitative measures of HLE were only assessed in half of the sample and therefore not considered in the analyses.*

### Measures

Descriptive statistics for all variables included are provided in Table 2. As the first research question refers to the proposed structure of qualitative and quantitative indicators of early HLE, we do not provide internal consistencies for these constructs in this section, but instead placed them in the “Results” section.

**TABLE 2:** Descriptives of HLE, parent-child courses, child’s vocabulary, and control variables.

	*n*	M (*SD*)/%	Min	Max
**Home Learning Environment**				
Joint picture book reading at 7 months	1013	3.13 (1.45)	1	5
Sensitivity at 7 months	1013	4.22 (0.72)	1	5
Stimulation at 7 months	1013	2.75 (0.92)	1	5
Positive affect at 7 months	1013	3.31 (0.92)	1	5
Prevalence of affect at 7 months	1013	2.73 (1.05)	1	5
Joint picture book reading at 26 months	1013	7.55 (0.79)	1	8
Visiting Library at 26 months	1013	2.37 (1.39)	1	8
Nursery Rhymes, Poems at 26 months	1013	6.38 (1.86)	1	8
Sensitivity at 26 months	1013	3.74 (0.78)	1	5
Language Stimulation at 26 months	1013	3.40 (0.77)	1	5
Prevalence of affect at 26 months	1013	3.54 (0.86)	1	5
**Parent-child–courses**				
Parent-child courses (0 = no 1 = yes)	1013	83%	0	1
**Child vocabulary**				
Child vocabulary at 38 months	1013	51.6 (26.90)	0	118
Child vocabulary at 26 months	1013	157.40 (57.00)	2	260
Child vocabulary at 13 months	968	2.15 (0.84)	1	5
**Indicators of family background (SES)**				
Maternal education (years of education)	1013	15.40 (2.29)	9	18
Income poverty (0 = no 1 = yes)	1013	11%	0	1
Single parent (0 = no 1 = yes)	1013	4%	0	1
**Controls**				
Child’s age (in months) at 38 months	1013	38.40 (0.95)	36	41
Child’s age (in months) at 26 months	1013	26.30 (0.98)	24	29
Child’s age (in months) at 7 months	1013	6.94 (0.80)	4	11
Child is a boy (0 = no 1 = yes)	1013	50%	0	1
Number of siblings	1013	0.83 (0.91)	1	7

#### Quantitative Dimension of the Home Learning Environment

The indicators of the quantitative dimension of the HLE was derived from an interview in which the parent (mostly the mother) was asked about the frequency of joint activities of the parent or other persons in the home with their child, adapted from other large scale longitudinal studies (EPPSE-study; Sylva et al., 2014). For the present study we used two time points, when the child was 7 (as control variable; see “Analytic Strategy”) and 26 months old. When the child was 26 months old, parents were asked how often they provide their child with nursery rhymes or songs, visit a library or bookstore together with the child, and read to the child / look at picture books. Parents indicated the frequency on an eight-point rating scale ranging from [1] never, [4] several times a month, to [8] several times a day. When the children were 7 months old, age-adjusted items were applied, from which we used the frequency of joint picture book reading (ranging from [0] never to [5] daily).

#### Qualitative Dimension of the Home Learning Environment

The quality indicators for the HLE were derived from an observed semi-standardized play situation conducted in the home of the family when the child was 7 (as control variable; see “Analytic Strategy”) and 26 months old (Linberg et al., 2019 for a description of the procedure). The measures were adapted from the NICHD Study of Early Child Care (NICHD Early Child Care Research Network, 1998). Parents and children were presented with a standardized toy set and parents were instructed to play with their child and the toys as they normally would. The videotaped interactions were rated afterwards by trained coders according to strict coding rules. The coding system was adapted from the NICHD study; parental as well as the child’s interaction behavior was rated on qualitatively defined 5-point scales, which captured to what extent the item was characteristic for the observed behavior of the parent/child, ranging from [1] not at all characteristic to [5] highly characteristic (NICHD Early Child Care Research Network, 1998; Linberg et al., 2019). Coders were extensively trained on the rating system and had to attain at least a 90% agreement (within 1-point) with “gold standard” ratings of videos before they were allowed to code independently. Inter-rater reliability was assured through randomly selecting 20% of the videos for double-coding. The average inter-rater agreement was above 90%.

The qualitative dimension of the HLE when children were 26 months old included three items capturing emotional support as well as stimulating interaction behavior. The item “Sensitivity to non-distress” indicates whether and how the parents respond to children’s signals of non-distress in a sensitive (prompt, contingent and appropriately warm) manner. The item “Language stimulation” assesses the amount and quality of verbal enrichment of the play situation, such as prompting and expanding child’s verbalizations, asking open ended questions, and verbal distancing. The item “Prevalence of affect” measures the dynamics of parental emotions, i.e., whether parents’ affect is flat (flat tone of vocal expression, impassive facial expression) or whether the parent shows emotions appropriate to the situation within the usual range. At age 7 months, the same but age-adapted procedure was administered, and the dimension consists of comparable but slightly age-adapted items including sensitivity, stimulation, positive affect, and prevalence of affect.

#### Parent-Child Courses

The indicator for “Attendance of parent-child courses” was derived from open questions within the parent interview. Parents were asked whether they attended any parent-child courses during the first 26 months of the child’s life. They could state as many courses as they liked (*M* = 1.82, *SD* = 1.32, Min = 0, Max = 5). Seventy nine percent of the parents attended parent-child courses with their child and most of them attended more than one course. The reported courses were mainly play or toddler groups (39%), but motoric- (30%), music-oriented courses (23%), and swimming (37%) were also reported. 30% of the parents attended registered trademark courses such as PEKiP or Accompanying Children’s Early Development (FenKid). We used the dichotomous indicator whether parents did (1) or did not (0) attend at least one parent-child course within the first 26 months of the child’s life. In doing so, we captured sheer course-attendance as we did not suppose a straight linear effect, meaning more courses resulting in an equally higher quality of HLE or larger vocabulary.

#### Vocabulary at 26 Months

Child’s (expressive) vocabulary at 26 months was assessed using a standardized German vocabulary check-list (“Elternfragebogen für zweijährige Kinder: Sprache und Kommunikation“(ELFRA-2); Grimm and Doil, 2006). This parent checklist is a German version of the internationally well-known “MacArthur Communicative Development Inventories (Toddler Form) – CDI” (Fenson et al., 1993) and the “Language Development Survey – LDS” (Rescorla and Achenbach, 2002). The ELFRA-2 contains 260 words, including nouns and verbs, for which the parents indicate whether the child already uses them actively (productive vocabulary). We used the sum of words actively used by the child as the indicator for children’s vocabulary. The validity of parent checklists and the ELFRA-2 has been assured through high correlations with standardized language tests (e.g., *r* = 0.78; of ELFRA-2 and the Reynell Developmental Language Scales III – RDLS-III (Edwards et al., 1997; Sachse et al., 2007); and expressive vocabulary has been shown to be more reliably accessible by parents than receptive vocabulary.

#### Vocabulary at 38 Months

At the age of 38 months child’s (receptive) vocabulary was tested via the German version of the Peabody Picture Vocabulary Test-Revision IV (Dunn and Dunn, 2007; Lenhard et al., 2015). The test was administered in the home of the children on a tablet computer on which the child had to tap on the one out of four pictures fitting to the orally presented word. A maximum of 19 sets with 12 items each were administered in this way and the task continued until the child got eight of 12 words incorrect in one section (LIfBi, 2019).

#### Indicators of Family Socio-Economic Background

We included three measures: risk of income poverty (0 = no; 1 = yes) was calculated for each family by identifying whether the equivalized household income lies below 60% of the median of nationwide equalized income (OECD, 2013). Living in a single parent household (0 = no; 1 = yes) indicates whether a partner lives in the household. Furthermore, maternal education as the sum of years of primary, secondary and tertiary education was considered.

#### Controls

Except for the confirmatory factor analyses we controlled for a standard set of family and child characteristics. These include child gender (1 = male), age of the child (in months) in the concurrent wave, number of siblings living in the household, and a rough proxy for children’s early vocabulary at the age of 13 months. Here, parents were asked how many things or persons the child already names correctly, so that the parent is able to understand the child, ranging from [1] none to [5] more than 20 persons or objects.

### Analytic Strategy

For analysing the structural relationship between the quantitative and qualitative dimension of the HLE, their association with family socio-economic background variables, parent-child courses and children’s vocabulary, we modeled the respective paths within structural equation modeling. To test for the structure of the dimensions of the early HLE, we modeled the frequency of language stimulating joint activities (quantitative dimension) as well as the quality of parent-child interaction (qualitative dimension) as two separate but associated aspects and tested this model against a competing unidimensional HLE model. Model fit was evaluated by chi-square, root mean square error approximation (RMSEA), and comparative fit index (CFI) (Hu and Bentler, 1999). We relied on the following benchmarks for assessing fit: RMSEA <0.05 signifies a good fit; CFI > 0.90 represents a good fit (Bollen, 1989; Hu and Bentler, 1999). In a second step we included indicators of family background, child’s vocabulary at the age of 26 and 38 months and parent-child courses to test for the relation between the early HLE and family background variables as well as children’s vocabulary development and the effect of parent-child-courses on the early HLE. To rule out the possibility that the expected indirect effect of attending parent-child courses via HLE is due to a confounded effect of very early HLE on vocabulary, or, in other words, that parents with high compared to lower early HLEs tend to more often attend parent-child courses, we simultaneously controlled in this model for quantitative and qualitative indicators of HLE at 7 months. Thus, HLE at 24 months represents an indicator of change in HLE. As mentioned before, we also controlled for child characteristics and family background.

All analyses were carried out using Stata 15. To deal with missing data, we chose the full information maximum likelihood (FIML) approach using valid information of all observations for model estimation (Enders, 2001; Acock, 2013).

## Results

**FIGURE 2:**
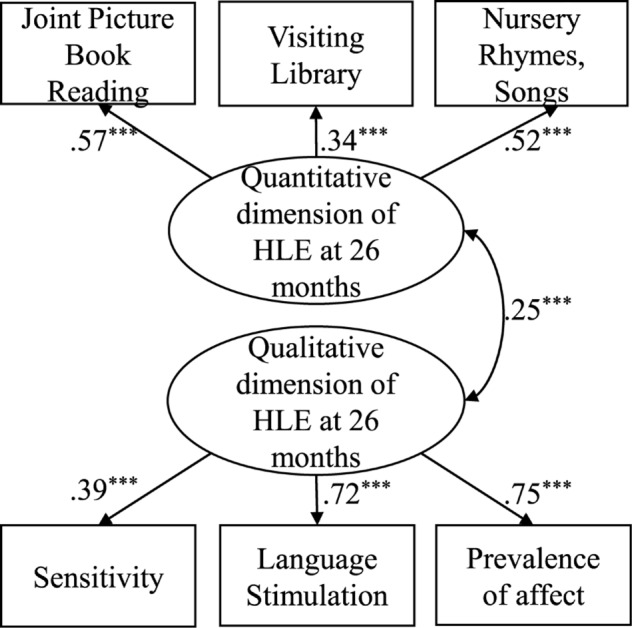
Two factor model of the structural relation between the qualitative and quantitative dimension of home learning environment. *N* = 1013; Standardized coefficients, ^+^*p* < 0.10, ^∗^*p* < 0.05, ^∗∗^*p* < 0.01, ^∗∗∗^*p* < 0.001; Model Fit: *χ*^2^(8) = 12.92, *p* < 0.01; RMSEA = 0.03; CFI = 0.99.

**FIGURE 3:**
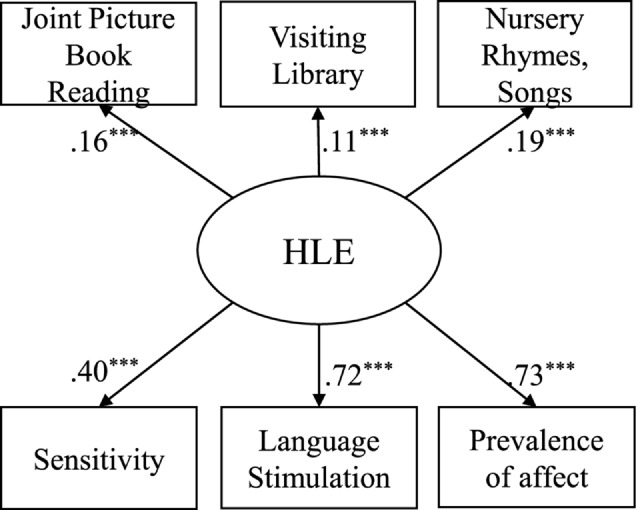
One factor model of the qualitative and quantitative indicators of home learning environment as unitary HLE construct. *N* = 1013; Standardized coefficients, ^+^*p* < 0.10, ^∗^*p* < 0.05, ^∗∗^*p* < 0.01, ^∗∗∗^*p* < 0.001; Model Fit: *χ*^2^(9) = 143.90, *p* < 0.001; RMSEA = 0.12; CFI = 0.79.

Results demonstrate that the frequency of joint activities and the quality of interactions are two separate aspects of the HLE in the early years that are moderately related (*r* = 0.25, *p*< 0.001; Figure 2). A comparative model, which considers only one latent variable demonstrates insufficient model fit [χ^2^(9) = 143.90 *p*<0.001; RMSEA = 0.12; CFI = 0.79; Figure 3].

**FIGURE 4:**
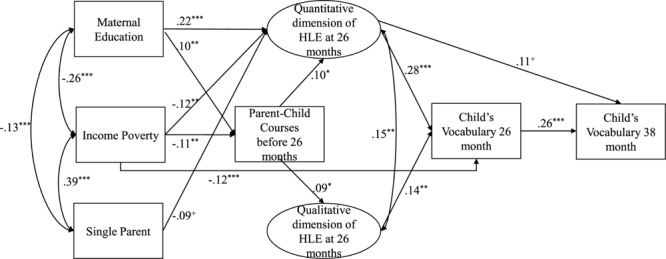
Relations of SES, the quantitative and qualitative dimension of the HLE and child’s vocabulary development with effects of parent-child courses. *N* = 1013; Standardized path coefficients, Only statistically significant paths are shown; ^+^*p* ¡ 0.10, ^∗^*p* ¡ 0.05, ^∗∗^*p* ¡ 0.01, ^∗∗∗^*p* ¡ 0.001; Model Fit: *χ*^2^(139) = 308.90, *p* < 0.001; RMSEA = 0.04; CFI = 0.94; R${}_{\mathrm{Quantity}\,\mathrm{HLE}}^{2}$ = 0.34; R${}_{\mathrm{Quality}\,\mathrm{HLE}}^{2}$ = 0.18; R${}_{26\,\mathrm{months}}^{2}$ = 0.28; R${}_{38\,\mathrm{months}}^{2}$ = 0.13; Covariates include child’s gender, age, siblings, child’s vocabulary at 13 months, quantitative and qualitative indicator of HLE at 7 months.

As shown in Table 3 and depicted in Figure 4, these dimensions of the HLE are differentially related to family socio-economic background variables. Whereas maternal education is significantly associated with the quantitative and qualitative dimension of HLE at age 7 months (Table 3), at 26 months (Table 3 and Figure 4), maternal education is still significantly connected to the change of the quantitative dimension of HLE; *r* = 0.22, *p* < 0.001.), but not to the change of quality of parent-child interaction (qualitative dimension of HLE). Moreover, being a single parent (*r* = −0.09, *p* < 0.10), as well as risk of income poverty (*r* = −0.12, *p* < 0.01), are significantly related to the change of quantity of stimulating activities of the HLE at 24 months though not to the change of the quality dimension (Figure 4). Income poverty, however, was also associated with the qualitative dimension of HLE at 7 months (Table 3). With regard to the relation of the HLE and children’s vocabulary development results indicate (Figure 4 and Table 3), even while controlling for the child’s vocabulary at 13 months, that the quantitative and the qualitative dimension of the HLE explain both unique variance of vocabulary development at the age of 26 months (*r* = 0.28, *p* < 0.001; *r* = 0.14, *p* < 0.001), respectively. At the age of 38 months, only the quantitative dimension adds to the effect of earlier vocabulary development on later receptive vocabulary (*r* = 0.11, *p* < 0.10; Figure 4 and Table 3).

**TABLE 3:** Standardized effects of the model on the relation between SES, the two dimensions of HLE and vocabulary development.

	Quantitative dimension of HLE at 7 months		Qualitative dimension of HLE at 7 months		Quantitative dimenaion of HLE at 26 months		Qualitative dimension of HLE at 26 months		Parent-child-courses		Child vocabulary at 26 months		Child vocabulary at 38 months	
	β	SE	β	SE	β	SE	β	SE	β	SE	β	SE	β	SE
Maternal education	0.08*	0.03	0.10**	0.03	0.22***	0.04	0.05	0.03	0.10***	0.03	0.01	0.03	0.04	0.03
Income poverty	–0.04	0.03	−0.09**	0.04	−0.12**	0.04	–0.05	0.03	−0.11***	0.03	−0.12***	0.03	–0.00	0.03
Single parent	0.02	0.03	0.05	0.04	−0.09^+^	0.04	–0.02	0.03	–0.02	0.03	–0.01	0.03	0.05	0.03
Child’s age	0.05	0.03	0.00	0.03	0.04	0.04	0.04	0.03	–0.03	0.03	0.18***	0.03	0.06*	0.03
Child is a boy	0.02	0.03	–0.05	0.03	−0.16***	0.04	–0.03	0.03	0.02	0.03	−0.06*	0.03	0.02	0.03
Number of siblings	–0.04	0.03	–0.05	0.03	–0.02	0.04	0.04	0.03	−0.21***	0.03	−0.08**	0.03	–0.02	0.03
Vocabulary – 13 months					0.16***	0.04	0.05	0.03	0.03	0.03	0.22***	0.03	–0.03	0.03
Vocabulary – 26 months													0.26***	0.03
Parent-child courses					0.10*	0.04	0.09*	0.04			–0.03	0.03	–0.03	0.03
Quantitative dimension of HLE at 7 months					0.31***	0.04	–0.04	0.04	0.06*	0.03	–0.00	0.04	0.00	0.04
Qualitative dimension of HLE at 7 months					0.15**	0.05	0.38***	0.04	0.01	0.03	–0.05	0.04	0.03	0.04
Quantitative dimension of HLE at 26 months											0.28***	0.06	0.11^+^	0.06
Qualitative dimension of HLE at 26 months											0.14**	0.04	0.04	0.04
*R*^2^	0.02		0.03		0.33		0.18		0.10		0.28		0.13	

N = 1013; Standardized coefficients, ^+^p < 0.10, *p < 0.05, **p < 0.01, ***p < 0.001.

When examining the effects of the attendance of parent-child courses (Figure 4 and Table 3), it can be seen that the two dimensions of HLE are both predicted by the attendance of parent-child courses, with similar effect sizes (*r* = 0.10, *p* < 0.001; *r* = 0.09, *p* < 0.05, respectively). Note that this effect is apparent even under the statistical control of the quantitative and qualitative dimension of the HLE at 7 months, which should reduce (and control for) the feasible effect that parents with comparatively higher early HLE or/and SES tend to attend more parent-child courses (see Table 3 for detailed results).

## Discussion

The first years of a child’s life are an important phase for stimulating children’s development, as this is a time of rapid change in different developmental areas. For children from the age of 3 years onwards it is well documented that they experience different and differentiated HLEs and that the kind, frequency, and quality of stimulating activities and interactions are at least partly associated with the socio-economic resources (such as income, education) of their families (Kluczniok et al., 2013). In addition, it has been shown that different dimensions of the HLE impact developmental progress in various areas of development differentially (Lehrl et al., 2020). With regard to vocabulary, many children fall behind their peers already at young ages (Brooks-Gunn and Markman, 2005; Dubowy et al., 2008; Hindman et al., 2008; Fernald et al., 2013). This is particularly challenging as vocabulary is linked to the development of receptive and productive oral language proficiency and reading skills and thus to opportunities for academic success and social participation. While these issues are extensively studied in children from the age of 4 years onwards, studies focusing on children in the very first years of their lives are comparatively sparse, especially those treating the HLE as a multi-dimensional environment. In addition, it is largely unclear to what extent low-threshold offers, such as parent-child courses, contribute to the strengthening of HLE at an early age and thus contribute to the child’s vocabulary development. Therefore, this paper examined: (1) the relation between different aspects of the early HLE, i.e., whether qualitative and quantitative aspects of stimulation are separable dimensions or whether they represent a unitary construct of the HLE, (2) whether they are differentially related to SES, and (3) whether attending parent-child courses contributes to enriched HLEs which in turn (4) predict the child’s vocabulary development between age two and three.

Our results indicate that quantitative and qualitative aspects of the early HLE should be differentiated. Although these aspects are slightly linked to each other, they do not share much common variance. Thus, an overall factor characterizing a more or less stimulating HLE did not account for the observed differences between HLEs. While only a few studies distinguished these process characteristics of the HLE in the very early years, the few studies that did so also indicate that a distinction is not only possible and meaningful from a theoretical perspective but also necessary to adequately describe different learning environments in the first years of life. Mistry et al. (2008) found that the quality of interactions between mother and child at age one and two was only slightly related to the frequency of literacy stimulation at the same age. Further, results from Linberg (2018) indicate that even more differentiated distinctions within the qualitative aspects of mother-child interactions are possible and necessary to explain environmental effects on child development. In particular, study results show that the differentiation of dimensions of learning environments, such as global and domain-specific stimulating and emotionally supportive aspects of the learning environment, might be important from early on (Leseman and de Jong, 1998; LeFevre et al., 2010; Lehrl et al., 2020). The differentiation of quantitative and qualitative aspects of the HLE also proves to be particularly useful as our analyses indicate that these dimensions tend to be specifically associated with the different indicators of socio-economic background of the family. As early as the age of 7 months, children experience a different quality in stimulation dependent on their mother’s educational level and household income, whereas the quantitative dimension (the frequency of activities) depends on maternal education only. All of the considered SES indicators, however, were associated with the (change) in the quantitative dimension of the HLE when children were about 2 years of age (however, different in extent) but not with the (change in the) qualitative dimension. Potentially the social and economic resources might be particularly relevant to changes in the frequency of stimulating activities, parents engage with their child, but less with the change of quality of interaction with their child (Lehrl, 2018). According to the family investment and stress model (Conger and Donnellan, 2007) single parents might lack of time for a high frequency of the considered activities, families suffering from economic hardship might lack the financial resources to provide children with stimulating materials that in turn offer chances for frequent stimulating activities and they might suffer from stress leading to emotional distress and as a consequence in non-functional parenting. However, it must be mentioned that relationships are rather weak. Somewhat more pronounced is the association of maternal education with the quantitative aspects of the HLE. This may be due to the fact that mothers with higher education tend to obtain more information on children’s development (Huang et al., 2005; Bornstein et al., 2010; Fagan, 2017) which in turn might lead to more frequent use of stimulating activities (such as reading more often to the child) rather than to a change of concrete interaction behavior (such as interacting more sensitively with a young child). However, it should not be prematurely concluded that SES does not affect the qualitative dimension at all. There is some evidence that the qualitative dimension may only be reduced when multiple SES-risk factors are cumulated (Mistry et al., 2010; Linberg, 2018). With regard to the question to what extent the two dimensions of the HLE are related to the development of children’s vocabulary results showed that, similar to existing research with older children (Bus et al., 1995; Mol et al., 2008; Flack et al., 2018) the HLE at a very early age is already related to the child’s vocabulary at the age of 2 years. In addition, our study results show that the dimensions are both associated with the development of children’s vocabulary at age two (although not as pronounced) when controlling for a proxy of early vocabulary, through adding both unique variance in early vocabulary development. Further, the relationship between the quantitative aspect and children’s vocabulary development between age two and three is additionally present, revealing stronger effects of the quantitative aspects, although both quantitative and qualitative aspects include language-promoting parenting behaviors. There is a very active discussion on the importance of quality vs. quantity of language-stimulating activities particularly with respect to shared book reading. Study results show that reading practices such as those realized in dialogic reading programmes are far more effective than verbatim reading to young children, asking closed yes-no questions or interactions characterized by a less frequent use of various language-teaching or distancing strategies (Whitehurst et al., 1988; Landry et al., 2012) that actively engage the child in the conversation. This points to the importance of the quality of these activities. Other studies, which partly use natural experiments, demonstrate that a sheer increase in joint reading is associated with advanced language skills (Barnes and Puccioni, 2017; Price and Kalil, 2019) which hints to the importance of frequency. However, note that we did not measure the quality of the activities we used for measuring the quantitative aspects of stimulation.

With regard to parent-child courses, our results indicate that sheer attendance of parent-child courses during the first 2 years of a child’s life seems to enrich the early HLE in both dimensions. This, albeit small, effect might be explained by the content of most parent-child courses, which often focus on stimulating activities as well as interactions during the course itself. This might stimulate parents to adopt these activities into their own everyday life at home.

However, we cannot completely rule out that the effect, which we interpret as an enrichment of the HLE by parent-child courses, results from the fact that parents with a higher HLE tend to attend parent-child courses more often. By controlling for the early HLE at the age of 7 months and socio-economic background characteristics, we account, to some degree, for this possibility, however, well designed intervention studies are needed to substantiate causal effects of attending parent-child-courses. Additionally we did not include potential moderators (such as emotional distress or social support) which might also drive the effect of parent-child-courses on HLE.

Concerning the reliability of the indicators of the HLE, it must be noted that although observational methods are considered as rather objective measures, the short observation time, which only observes one play situation, might not give a full picture of the quality of interactions at home. Although, Vogel et al. (2015) have demonstrated that the mother’s interaction behavior assessed with the same instrument in a play situation is significantly correlated with the interaction behavior in other situations, this rather short impression of the quality of the HLE, might explain why the association found between HLE and child’s vocabulary is rather low in effect size.

Another limitation of the present study is the missing information on the specific content of the parent-child courses or their quality, and the resulting missing classification according to content areas. Research results for studies on intervention programs, however, point to the importance of the quality of these courses (Cadima et al., 2017). Thus, when investigating the effects of such interventions research should consider content and aspects of quality of such courses.

## Conclusion

Research on improving language skills and reducing SES-related disparities in acquiring language skills is an area of strong interest for researchers, educators, and policy makers, as it lays the basis for effectively promoting long-term educational success for all children. Variations in the HLE have therefore been of major research interest in the last several decades. The results of the present study show that the HLE, even at this very young age of 1 and 2 years old children, can be differentiated according to quantitative and qualitative aspects, that those aspects vary as a function of socio-economic family background, and that they predict vocabulary development in children. Furthermore, the present study reveals that attending low-threshold courses that aim at providing parents with information on nutrition, play, and motor development, and that at the same time enable exchanges with other parents, is positively associated with the HLE dimensions which in turn are predictive for child vocabulary development.

Practitioners who work with parents or parent-child-dyads should be aware of the various aspects of the HLE that affect children’s development, including the frequency of joint activities, e.g., shared book reading, the quality of parents’ interactions with children during such activities, and, although not studied in the present study, the materials that parents provide to their children to stimulate learning. Additionally, parent-child-courses, which are cost- and time-efficient and have a low threshold for participation, might be a possibility to approach the enrichment of the HLE in the very early years. Making these courses more accessible to particularly low SES families could be a way of addressing social disparities from the early on. Although, the effects are hardly comparable to elaborate programmes, parent-child courses might be cost- and time-efficient to initiate changes for a wider low-SES public. However, if and to what extent unequal starting conditions can be compensated by low-threshold courses and which elements of those courses are particularly meaningful for enriching the HLE are questions for future research.

## Data Availability Statement

Data from the National Educational Panel Study (NEPS): Starting Cohort Newborns, 10.5157/NEPS:SC1:6.0.0 which were used in this study are available as scientific use file. See http://www.neps-data.de.

## Ethics Statement

Ethical review and approval was not required for the study on human participants in accordance with the local legislation and institutional requirements. Written informed consent from the participants’ legal guardian/next of kin was not required to participate in this study in accordance with the national legislation and the institutional requirements.

## Author Contributions

AL organized the database and performed the statistical analysis. SL wrote the first draft of the manuscript. All authors wrote the sections of the manuscript, contributed to the design, the idea of the study, the manuscript revision, and read and approved the submitted version.

## Conflict of Interest

The authors declare that the research was conducted in the absence of any commercial or financial relationships that could be construed as a potential conflict of interest.
